# TRPV1 Receptor-Mediated Hypoglycemic Mechanism of Capsaicin in Streptozotocin-Induced Diabetic Rats

**DOI:** 10.3389/fnut.2021.750355

**Published:** 2021-10-06

**Authors:** Shiqi Zhang, Lanlan Tang, Fanshu Xu, Yonghai Hui, Hongjia Lu, Xiong Liu

**Affiliations:** ^1^College of Food Science and Engineering, Lingnan Normal University, Zhanjiang, China; ^2^College of Food Science, Southwest University, Chongqing, China; ^3^Department of Cell and System Biology, Faculty of Arts and Science, University of Toronto, Toronto, ON, Canada; ^4^College of Landscape Architecture and Life Science/Institute of Special Plants, Chongqing University of Arts and Science, Chongqing, China

**Keywords:** capsaicin, TRPV1, glycometabolism, blood glucose, insulin secretion, hypoglycemic effect

## Abstract

Our previous research showed that capsaicin exhibits hypoglycemic effects by activating the transient receptor potential vanilloid 1 (TRPV1) channel in diabetic rats. Interestingly, capsiate was also able to activate the TRPV1 channel, but with a non-significant hypoglycemic effect. This study aimed to investigate the effect of capsaicin on the glycometabolism of streptozotocin (STZ)-induced diabetic rats by blocking the TRPV1 channel. After a 4-week capsaicin treatment (6 mg/kg·bw), the serum insulin level of STZ-induced diabetic rats increased from 15.2 to 22.1 mIU/L, the content of hepatic glycogen and muscle glycogen increased by 81.2 and 20.2%, respectively, and the blood glucose level decreased significantly from 19.3 to 14.7 mmol/L. When the TRPV1 channel was blocked, capsaicin lost the above-mentioned effects, and the hypoglycemic effect was no longer significant. It was concluded that a combined up-regulation of both TRPV1 receptors and pancreatic duodenal homeobox-1 (PDX-1) led to the hypoglycemic effect of capsaicin, which partially explains our previous observation: capsiate activating TRPV1 without showing a significant hypoglycemic effect was due to the lack of a significant up-regulation of PDX-1. Based on the experimental results, we speculated that two signaling pathways [TRPV1-(PDX1)-(GLUT2/GK) and TRPV1-(PDX-1)-(IRS1/2)] exist in the pancreas of STZ-induced diabetic rats.

## Graphical Abstract

Signal pathway of hypoglycemic mechanism of capsaicin in the pancreas of STZ-induced diabetic rats. 
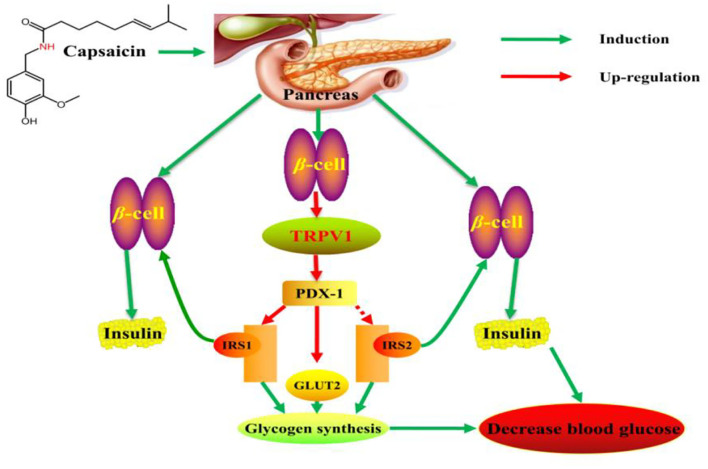

## Introduction

Diabetes is a metabolic disorder caused by insufficient insulin secretion, action, or resistance (1–3). It is widely recognized as one of the leading causes of death and disability in the Western world. With the rising incidence of diabetes, capsaicin (8-methyl-N-vanillylnonanamide), a phytochemical present in chili peppers, has attracted much attention due to its hypoglycemic effects (4, 5). Studies have shown that capsaicin can reduce fasting blood glucose levels in obese mice and improve glucose tolerance by increasing the insulin content (6). Furthermore, capsaicin treatment can also stimulate the secretion of insulin from rat islet cells by activating the transient receptor potential vanilloid 1 (TRPV1) ion channel, thereby achieving a hypoglycemic effect (7). In addition, capsicum has long been used in the treatment of diabetes in traditional Jamaican medicine, and capsaicin has also been used in the treatment of diabetic neuropathy (8, 9). However, the related mechanism of action is still unclear. The proposed hypoglycemic mechanism of capsaicin is through the activation of the TRPV1 channel, which promotes intracellular Ca^2+^ concentrations and leads to the activation of further downstream pathways (10–12). Moreover, a large number of studies have shown that TRPV1 has the function of distinguishing and integrating various pain stimuli, and is involved in the regulation of anti-inflammatory, analgesic and weight-reducing mechanisms (13–15). Conversely, some studies have shown that the above effects of capsaicin are not related to the activation of the TRPV1 channel (16, 17). Our previous research showed that the hypoglycemic effect of capsaicin was associated with activation of the TRPV1 ion channel and up-regulation of TRPV1 receptor expression in the liver and pancreas (18). Capsiate is equally capable of activating the TRPV1 ion channel, but no significant hypoglycemic effect has been observed (19). Therefore, the role of TRPV1 in the hypoglycemic effect of capsaicin requires further investigation.

In this study, capsazepine was used to block the TRPV1 channel in order to examine the role of TRPV1 receptors in the hypoglycemic mechanism of capsaicin. Streptozotocin (STZ)-induced diabetic rats were treated with an intraperitoneal injection of capsazepine and oral administration of capsaicin. Relevant indicators of hypoglycemia were measured, including the apparent absorptivity of total sugar, blood glucose level, oral glucose tolerance, plasma insulin level, glycogen content, and glycometabolism-related genes and protein expression levels in the liver, pancreas and ileum.

## Materials and Methods

### Materials

Four-week-old male Sprague-Dawley (SD) rats were used in the present study, and a standard diet was purchased from Chongqing Tengxin Inc. (Chongqing, China).

### Chemicals

Capsaicin (95% purity) and STZ were purchased from Sigma-Aldrich (St. Louis, MO). Capsazepine (98% purity) was purchased from Shanghai Hanxiang Biotechnology Co., Ltd (Shanghai, China). Glycogen and insulin (INS) enzyme-linked immune detection kits were purchased from Nanjing Jiancheng Bioengineering Institute (Nanjing, China). Amylase was supplied by Zhengzhou Tianle Chemical Products Co., Ltd. (Zhengzhou, China). Other chemical reagents were of analytical grade and purchased from Dishui Chemical Co., Ltd. (Chongqing, China).

### Capsaicin and Capsazepine Solution Preparation

(1) We weighed and dissolved 0.5 g of capsazepine in a 50 mL mixed solution of tween 80 (2%), ethanol (2%) and normal saline (96%) (20). Capsazepine was stored at 4°C prior to use, and a 10 mg/mL capsazepine peritoneal injection was prepared. Ultrasound assisted dissolution was performed prior to injection.

(2) Capsaicin was weighed according to the tolerance dose (2 mL/kg) by gavage in rats. After being mixed with soybean oil via ultrasonication and configured with a concentration of 3 mg/mL, the prepared capsaicin solution was stored for use at 4°C.

### Diabetes Induction and Experimental Design

Four-week-old male SD rats were maintained under controlled conditions (25 ± 1°C, 55 ± 5% relative humidity, and a 12-h light/dark cycle). Distilled water and solid feed were given *ad libitum*. After 1 week of feeding, diabetic rats induced by STZ (60 mg/kg, intraperitoneal injection) and the control group rats were injected with the same dose of citrate buffer. Fasting blood glucose (FBG) levels were measured by tail blood sampling after 72 h, and rats with FBG ≥ 11.1 mmol/L were screened as diabetic rats and underwent the capsaicin treatment experiment. Experimental rats were divided into five groups (8 rats/group): control group (healthy group), model group (hyperglycemic, no treatment), and three diabetes treatment groups [capsazepine group (CPZ), capsaicin group (Cap), and capsaicin and capsazepine group (Cap+CPZ)]. The control and model groups were lavaged with 2 mL/kg·bw soybean oil and administered 1 mL/kg·bw blank solvent [tween 80 (2%), ethanol (2%), and normal saline (96%)] by intraperitoneal injection; the CPZ group was lavaged with 2 mL/kg·bw soybean oil and 10 mg/kg·bw capsazepine by intraperitoneal injection 10; the Cap group was lavaged with 6 mg/kg·bw capsaicin and 1 mL/kg·bw blank solvent [tween 80 (2%), ethanol (2%), and normal saline (96%)] by intraperitoneal injection; the Cap+CPZ group was intraperitoneally injected with 10 mg/kg·bw capsazepine and lavaged with 6 mg/kg·bw capsaicin. In addition, an intraperitoneal injection of capsazepine and gavage capsaicin was conducted at 9 a.m. every day for 4 weeks. All of the rats had common feedstuff and drank water freely, and the control and model groups were treated with the same amount of soybean oil.

Animal experiments were conducted following the rules of the International Animal Welfare Committee Requirements and Regulations. The experiments were ethically acceptable and approved by the Committee on Animal Experimentation [experimental animal license SCXK (Chongqing) 200120008].

### Sample Collection

This animal experiment lasted 4 weeks; the food intake was measured every 2 days, and fresh feces were collected from rats in each group on the last 2 days of the experiment. Blood samples were taken from the necks of the rats, and plasma was separated via centrifugation at 1,400 rpm and 4°C for 15 min and then stored at −80°C. The quadriceps, liver, pancreas, and ileum were isolated, and samples were snap frozen and stored at −80°C for subsequent RNA isolation and gene expression analysis (19, 21). In addition, the remaining pancreatic tissue was taken for HE staining, and the pancreatic section was observed and photographed using Olympus BX53 microscope and DP80 high-resolution digital imaging system.

### Biochemical Index and Determination of Apparent Absorptivity of Total Sugar

Feces were collected from rats in every group on the last 2 days of the experiment. The fodder and feces samples were pretreated by amylase hydrolysis for 2 h at 70°C (18), and then the total sugar (TS) was measured in the fodder and feces using the anthrone colorimetry method (the formula is as follows) (22). Biochemical indicators such as insulin, glycosylated serum protein (GSP), and hepatic and muscle glycogen were determined using relevant reagent kits (Nanjing Jiancheng Bioengineering Institute, Nanjing, China). An oral glucose tolerance test (OGTT) was conducted according to the method described by Yu-Ming You (21).


$$\begin{array}{l} \text{Digestibility of TS }(\%) =100\text{-}100\times \;\left(\frac{\;\%\text{TS in fodder}}{\;\%\text{TS in feces}}\right) \end{array}$$

### RNA Extraction and Quantitative RT-PCR Analysis

The total RNA was extracted from the frozen tissue samples (liver, pancreas, and ileum) according to the method described by You et al. (21). The concentration and purity of RNA were quantified using a NanoDrop 1000 spectrophotometer (Thermo Scientific, Delaware, USA), and the integrity of RNA was verified by agarose gel electrophoresis using a Gel Doc XR^+^ System (Bio-Rad, Hercules, CA, USA). Subsequently, 2 μg of RNA was reverse transcribed to cDNA using a PrimeScript RT Reagent Kit (TaKaRa Bio, Otsu, Japan). The mRNA expression of liver X receptor (LXR), glucose transporter 2 (GLUT2), glucose transporter 5 (GLUT5), pancreatic duodenal homeobox-1 (PDX-1), sodium-glucose cotransporter 1 (SGLT1), glucose 6 phosphatase (G6pase), glucokinase (GK), phosphoenolpyruvate carboxykinase (PEPCK), insulin receptor substrate 1 (IRS1), insulin receptor substrate 2 (IRS2), and TRPV1 were determined using RT-PCR with a light cycler instrument (Roche Diagnostics, Mannheim, Germany). A total of 2 μL of cDNA and 10 μL of SYBR Premix Ex Taq II (Bio-Rad Corp., USA) were freshly mixed before the experiment. The polymerase activation and DNA were initially incubated at 95°C for 30 s, followed by 40 cycles of denaturation at 95°C for 5 s and then 60°C for 30 s. The 2^−ΔΔCT^ method was used to calculate the relative expression level of each gene, and the beta-actin gene was used as the reference (19, 21).

### Western Blot Analysis of the Target Proteins

Western blot analyses of LXR, PEPCK, G6Pase, GK, PDX-1, GLUT2, TRPV1, SGLT1, GLUT5, IRS1, and IRS2 were performed using standard procedures as described by Zhang et al. (19) and You et al. (21). PVDF membranes were purchased from Millipore Co., Ltd. (Billerica, MA, USA), and all of the primary antibodies were purchased from Abcam Inc. (Cambridge, MA, USA).

### Statistical Analysis

All data were expressed as means and standard deviations (*n* = 8). Data were subjected to a one-way analysis of variance using Origin 8.5 and SPSS version 19.0. The differences among groups were examined by Duncan's multiple-range test. *P* < 0.05 was considered statistically significant.

## Results

### Apparent Absorptivity of Total Sugar, Body Weight, and Food Intake

As shown in Table 1, no significant differences were observed in the food intake or body weight of rats before the treatment, while after 28 days of feeding, there were significant differences in the food intake, sugar intake and fecal excretion among each group. The content of TS in feces increased significantly in the Cap group, and the apparent absorptivity of TS in the Cap group was reduced by 1.84 and 1.83% compared to the model and CPZ groups, respectively. Meanwhile, the apparent absorptivity of the Cap+CPZ groups showed a 0.30 and 0.31% increase compared to the model and CPZ groups, respectively.

**Table 1:** Effects of TRPV1 receptor antagonist and capsaicin on weight and apparent absorption of TS in STZ-induced diabetic rats.

**Project**	**Control**	**Diabetes groups**			
		**Model**	**CPZ**	**Cap**	**Cap+CPZ**
Food intake (g/d)	26.2 ± 1.93	**46.3** **±** **2.07**^**a**^	43.5 ± 1.28^b^	34.7 ± 1.00^c^	39.8 ± 1.58^d^
Sugar intake (g/d)	12.7 ± 0.93	**22.4** **±** **1.00**^**a**^	21.0 ± 0.62^b^	16.8 ± 0.48^c^	19.2 ± 0.76^d^
Fecal excretion (g/d)	2.83 ± 0.19	**4.99** **±** **0.23**^**a**^	4.52 ± 0.16^b^	3.77 ± 0.10^c^	4.32 ± 0.17^d^
Sugar in feces (%)	27.4 ± 3.47	**36.8** **±** **6.00**^**a**^	35.1 ± 3.78^a^	58.6 ± 2.65^b^	25.9 ± 1.09^c^
Sugar excretion (g/d)	0.78 ± 0.15	**1.85** **±** **0.38**^**a**^	1.59 ± 0.20^b^	2.21 ± 0.13^c^	1.12 ± 0.05^d^
Apparent absorptivity (%)	97.8 ± 0.17	**98.3** **±** **0.34**^**a**^	98.3 ± 0.17^a^	96.5 ± 0.21^b^	98.6 ± 0.10^c^
Body weight (0 day)	265 ± 7.42	**264** **±** **7.96**	260 ± 18.09	261 ± 15.26	260 ± 17.48
End body weight (28 day)	324 ± 14.31	**275** **±** **7.40**	275 ± 17.10	288 ± 16.11	280 ± 13.78
Body weight gain (g)	59.9 ± 16.70	**11.1** **±** **3.27**^**a**^	15.4 ± 5.15^a^	27.1 ± 8.84^b^	19.6 ± 7.09^ab^

*All data are expressed as means ± SD, n = 8. Bold means are significant difference compared with the control group (P < 0.05). Means with different superscript letters are significantly different among the diabetes groups (P < 0.05). Effective concentrations of capsaicin and capsazepine are 6 and 10 mg/kg·bw, respectively*.

The effect of capsaicin on the body weight of STZ-induced diabetic rats is shown in Table 1. Compared to the control group, the model and CPZ groups showed significantly lower weight gain. Compared to the model and CPZ groups, the weight of rats in the Cap group significantly increased, by 2.44 and 1.76 times, respectively. The Cap+CPZ group only increased by 1.76 and 1.28 times when compared to the model and CPZ groups, respectively, but the differences were non-significant.

### Fasting Blood Glucose and Oral Glucose Tolerance Test

**Figure 1:**
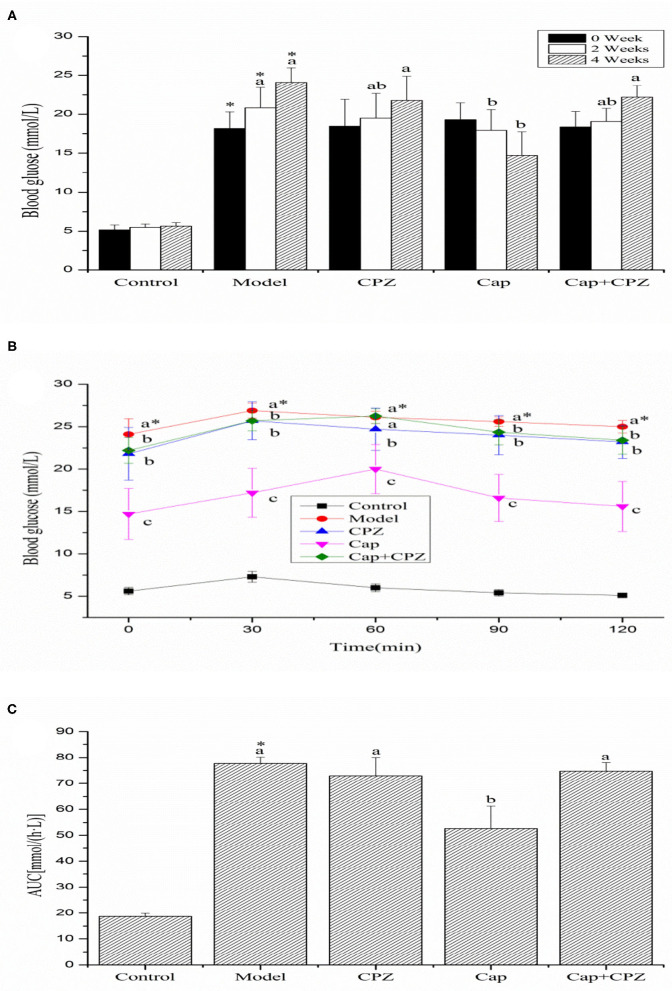
Effects of TRPV1 receptor antagonist and capsaicin on the oral glucose tolerance test (OGTT) **(A)**, area under curve (AUC) **(B)**, and fasting blood glucose **(C)** in STZ-induced diabetic rats. All data are expressed as means ± SD, *n* = 8. ^*^Means are significant difference compared with the control group (*P* < 0.05). Means with different superscript letters are significantly different among the diabetes groups (*P* < 0.05). Effective concentrations of capsaicin and capsazepine are 6 and 10 mg/kg·BW, respectively.

As shown in Figure 1A, no significant differences were observed in the blood glucose levels of the diabetes groups before the treatment (0 week). Compared to the normal control group, the blood glucose level (BGL) of the model and CPZ groups significantly increased. The hypoglycemic effects of capsaicin became more pronounced with each time increment following the feeding. A significant reduction (23.42%) in the BGL of the Cap group was observed after 4 weeks. In contrast, the BGL of the Cap+CPZ group showed a significant increase.

As shown in Figures 1B,C, the trend observed during OGTT was a peak in blood glucose at 30 min in the control, model and CPZ groups, while the peak in the Cap and Cap+CPZ groups was delayed by 30 min. We observed that after the four groups reached their peak blood glucose level (BGL), the rate of decline was fastest in the Cap group, and the areas under the blood glucose concentration curve showed significant differences (Figure 1B). Conversely, the rate of decline of the Cap+CPZ group was slow compared with the Cap group, and the areas under the blood glucose concentration curve were not remarkably different.

### Determination of Serum Insulin, GSP, and Glycogen

**Figure 2:**
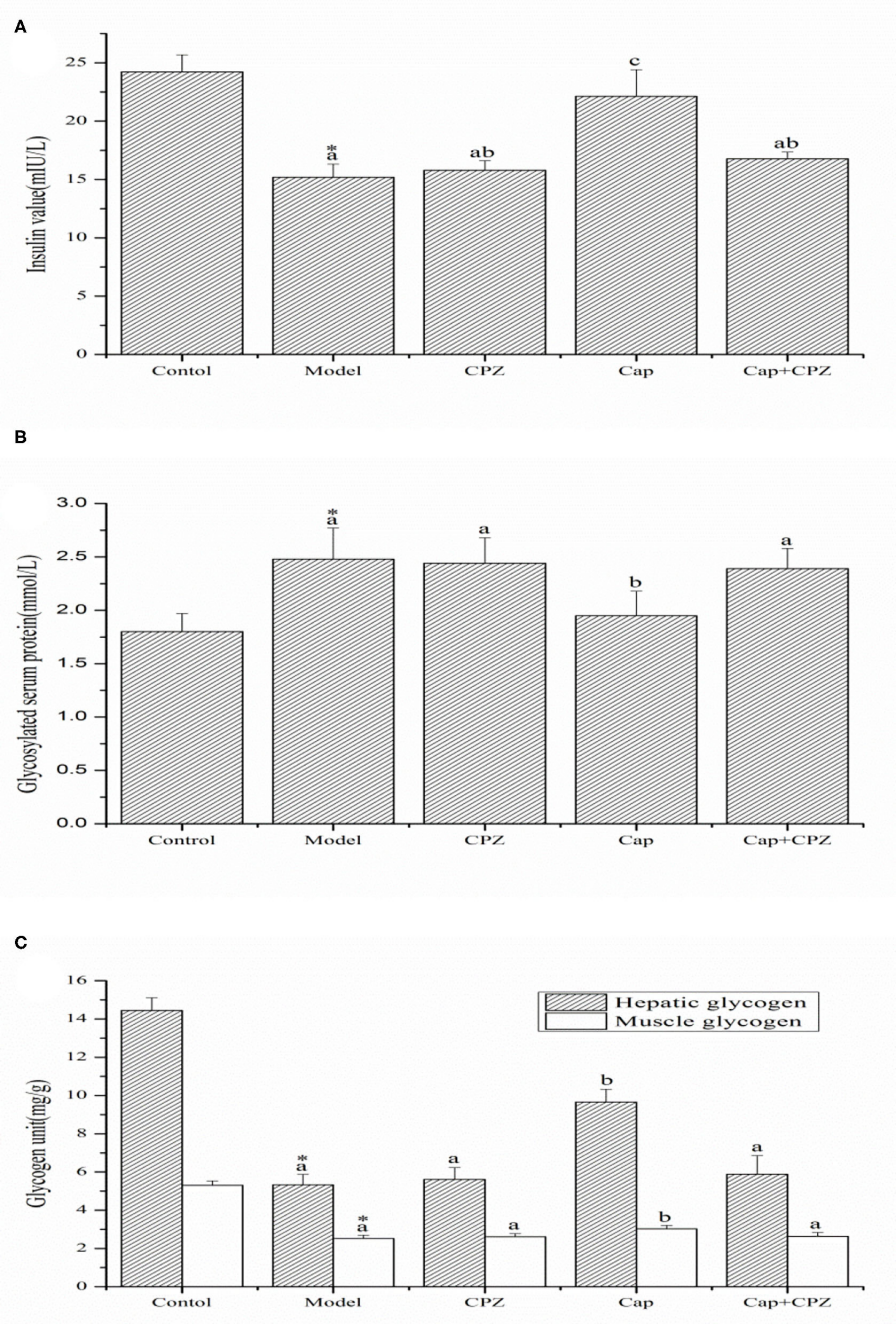
Effects of TRPV1 receptor antagonist and capsaicin on the serum insulin **(A)**, glycosylated serum protein **(B)**, and hepatic glycogen and muscle glycogen **(C)** in STZ-induced diabetic rats. All data are expressed as means ± SD, *n* = 8. ^*^Means are significant difference compared with the model group (*P* < 0.05). Means with different superscript letters are significantly different among the diabetes groups (*P* < 0.05). Effective concentrations of capsaicin and capsazepine are 6 and 10 mg/kg·BW, respectively.

As shown in Figure 2A, the serum insulin content in the model group was significantly lower compared to the control group. After 4 weeks of capsaicin gavage and capsazepine intraperitoneal injection, serum insulin values in the Cap group (22.13 ± 2.28 mIU/L) increased significantly, by 50.5% compared to those in the model group (15.2 ± 1.13 mIU/L), while the model and Cap+CPZ groups showed different degrees of increase, although the differences were non-significant.

As shown in Figure 2B, compared with the control group, glycosylated serum protein in the model group significantly increased. After treatment with capsaicin and capsazepine, glycosylated serum protein (1.95 ± 0.23 mmol/L) in the Cap group significantly decreased by 21.4% compared to the model group (2.48 ± 0.29 mmol/L), while the CPZ and Cap+CPZ groups showed different degrees of non-significant decline.

Liver glycogen and muscle glycogen in the model group were significantly reduced compared to the control group, as shown in Figure 2C. After treatment with capsaicin and capsazepine, the glycogen content of the Cap group increased significantly compared with that of the model group, and the content of hepatic glycogen and muscle glycogen increased by 81.2 and 20.2%, respectively, while the CPZ and Cap+CPZ groups showed different degrees of increase, although the differences did not reach significance.

### Effects of TRPV1 Receptor Antagonist and Capsaicin on Pancreatic Tissue Morphology in STZ-Induced Diabetic Rats

**Figure 3:**
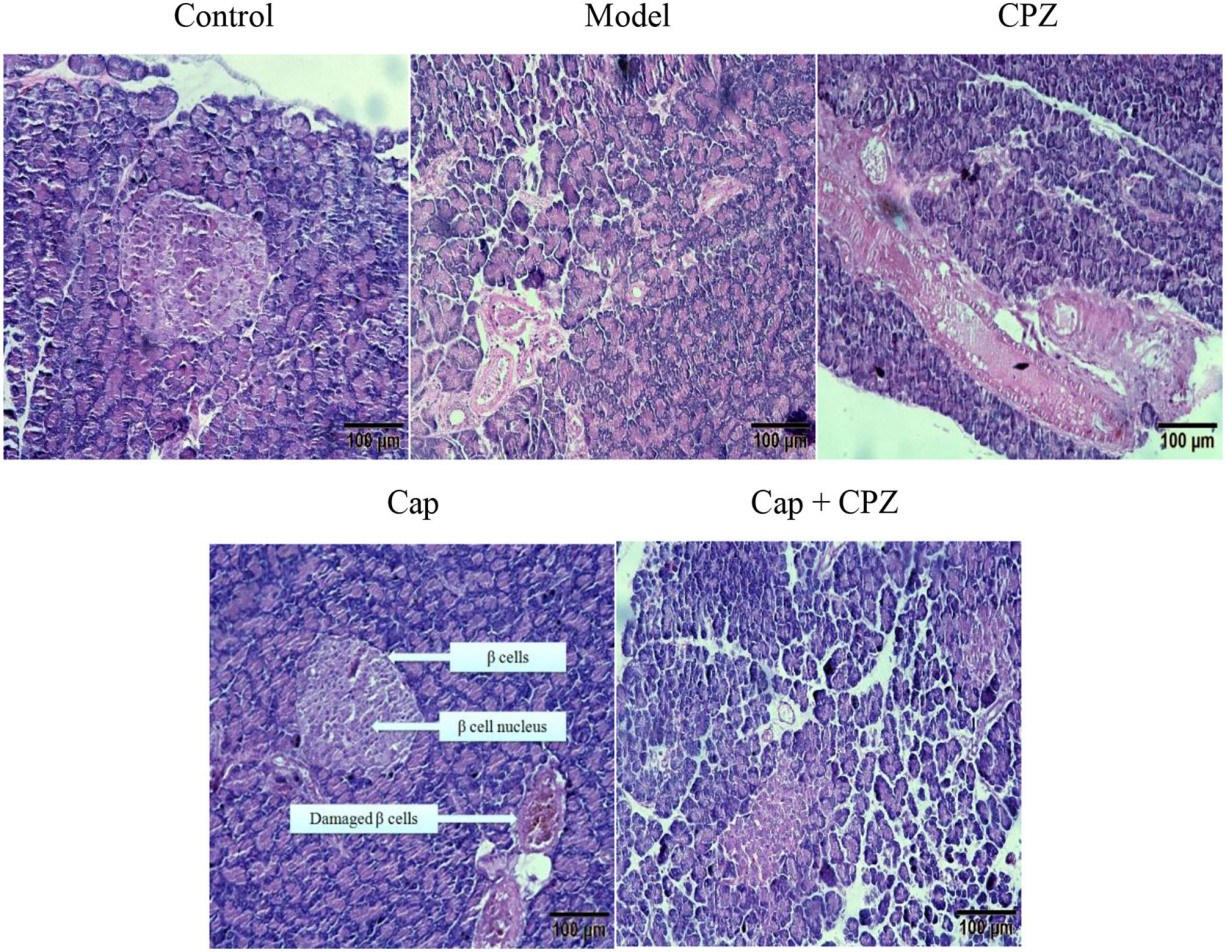
Effects of TRPV1 Receptor Antagonist and Capsaicin on pancreatic tissue morphology in STZ-induced diabetic rats. Pancreatic tissue sections were stained with HE and photographed with a scale of 100 μm. Effective concentrations of capsaicin and capsazepine are 6 and 10 mg/kg·BW, respectively.

As shown in Figure 3, in the control group, the pancreatic tissue structure was arranged regularly, the islet cells were evenly distributed in the pancreatic tissue, and the β cells and nuclei (black dots) were clearly visible. The situation of model group and CPZ group was similar, with disorderly arrangement of islet structure and uneven distribution of β cells. After 4 weeks of capsaicin treatment, the islet tissues were evenly arranged, and β cells and nuclei were clearly seen. In Cap+CPZ group, islet tissue was sparse, and β cells were still in pathological state with fuzzy boundary. These results indicate that capsaicin can improve the damage of streptozotocin on pancreatic and islet beta cells, and has a certain repair effect on the damaged pancreas. Capsazepine not only has no improvement effect on pancreatic tissue and islet beta cells, but also can inhibit the improvement of capsaicin on islet beta cells.

### Effects of TRPV1 Receptor Antagonist and Capsaicin on mRNA and Protein Levels of Key Genes for Glycometabolism in Liver of STZ-Induced Diabetic Rats

**Figure 4:**
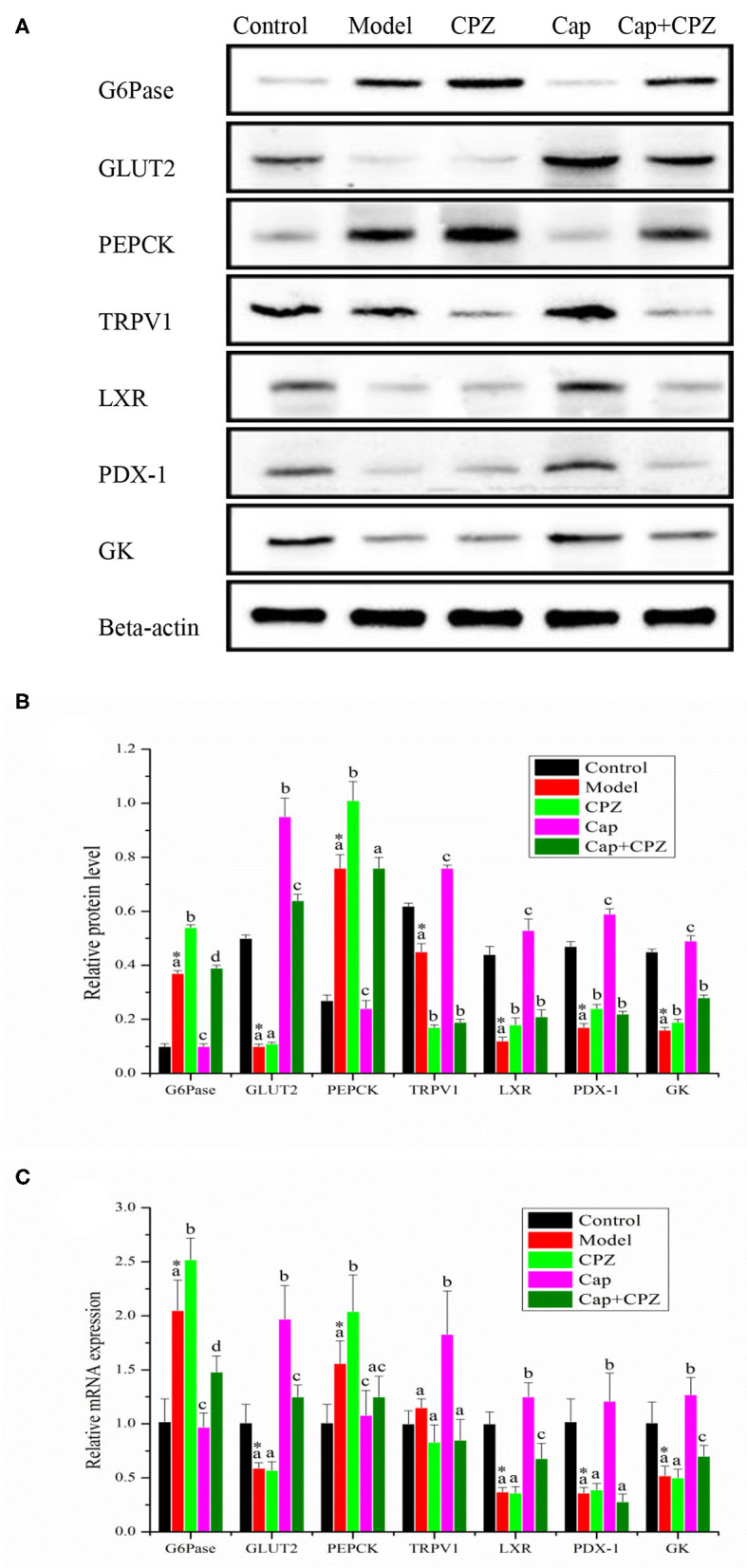
Effects of TRPV1 receptor antagonist and capsaicin on protein **(A,B)** and mRNA **(C)** expression levels of key genes for glycometabolism in liver of STZ-induced diabetic rats. All data are expressed as means ± SD, *n* = 8. ^*^Means are significant difference compared with the control group (*P* < 0.05). Means with different superscript letters are significantly different among the diabetes groups (*P* < 0.05). G6pase, glucose 6 phosphatase; GLUT2, glucose transporter 2; PEPCK, phosphoenolpyruvate carboxykinase; TRPV1, transient receptor potential cation channel subfamily V member 1; LXR, liver X receptor; PDX-1, pancreatic duodenal homeobox 1; GK, glucokinase. Effective concentrations of capsaicin and capsazepine are 6 and 10 mg/kg·BW, respectively.

As shown in Figures 4A–C, compared with the control group, the mRNA expression and protein levels of GLUT2, LXR, PDX-1, and GK in the model group were significantly down-regulated, and G6pase and PEPCK were significantly up-regulated. For the Cap group, the mRNA expression and protein levels of GLUT2, PDX-1, GK, LXR, and TRPV1 were significantly up-regulated compared to the model group, while LXR, PDX-1, and GK were up-regulated to different extents in the CPZ group, but with no significant differences. In the Cap+CPZ group, the mRNA expression and protein levels of GLUT2, LXR, and GK were significantly up-regulated, and G6pase was significantly down-regulated, but the changes were not as significant as those in the Cap group. These results show that the blocking effect on the TRPV1 ion channel by capsazepine indirectly weakened the effect of capsaicin on each gene. In addition, compared to the model group, the expression of TRPV1 in the CPZ group was significantly down-regulated, indicating that capsazepine can not only block the TRPV1 ion channel, but also affect the expression of TRPV1 receptors.

### Effects of TRPV1 Receptor Antagonist and Capsaicin on mRNA and Protein Levels of Key Genes for Glycometabolism in Pancreas of STZ-Induced Diabetic Rats

**Figure 5:**
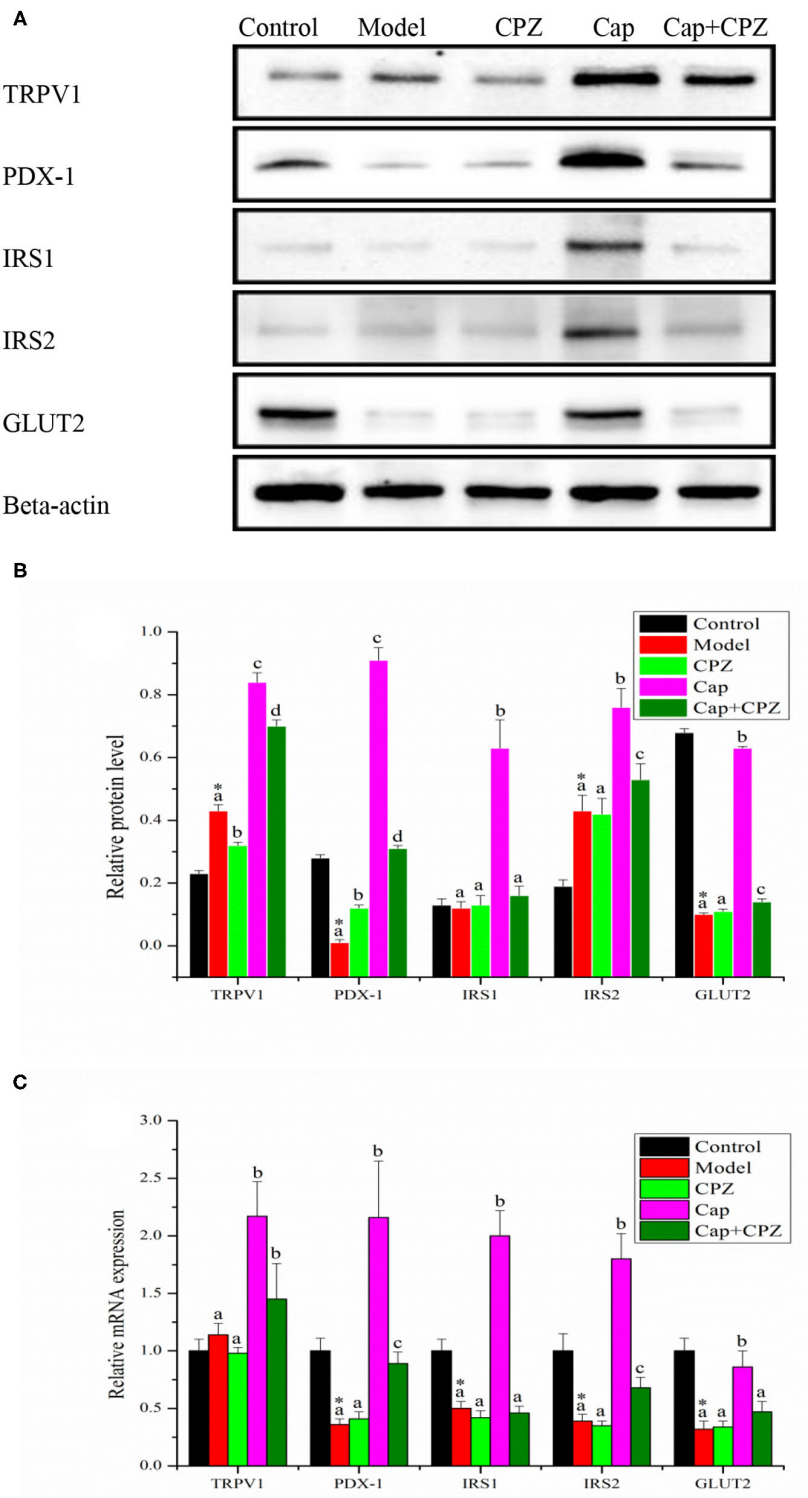
Effects of TRPV1 receptor antagonist and capsaicin on protein **(A,B)** and mRNA **(C)** expression levels of key genes for glycometabolism in pancreas of STZ-induced diabetic rats. All data are expressed as means ± SD, *n* = 8. ^*^Means are significant difference compared with the control group (*P* < 0.05). Means with different superscript letters are significantly different among the diabetes groups (*P* < 0.05). TRPV1, transient receptor potential cation channel subfamily V member 1; PDX-1, pancreatic duodenal homeobox-1; IRS1, insulin receptor substrate 1; IRS2, insulin receptor substrate 2; GLUT2, glucose transporter 2. Effective concentrations of capsaicin and capsazepine are 6 and 10 mg/kg·BW, respectively.

As shown in Figures 5A–C, compared with the control group, the mRNA expression and protein levels of PDX-1 and GLUT2 were significantly down-regulated in the model group. Compared with the model group, the mRNA expression and protein levels of TRPV1, PDX-1, IRS1, IRS2, and GLUT2 were significantly up-regulated in the Cap group; in the CPZ group, the mRNA expression of PDX-1 was significantly up-regulated, but with no significant difference in the protein levels, and the mRNA expression and protein levels of IRS1, IRS2, GLUT2 showed no significant differences; in the Cap+CPZ group, the mRNA expression and protein levels of TRPV1, PDX-1, and IRS2 were significantly up-regulated, but not as significantly as those in the Cap group. In addition, compared with the control group, an interesting phenomenon was observed, which is that the protein levels of IRS2 were significantly up-regulated in the model group, and we speculate that this may be due to the self-regulation of the rat's immune system.

### Effects of TRPV1 Receptor Antagonist and Capsaicin on mRNA and Protein Levels of Key Genes for Glycometabolism in Ileum of STZ-Induced Diabetic Rats

**Figure 6:**
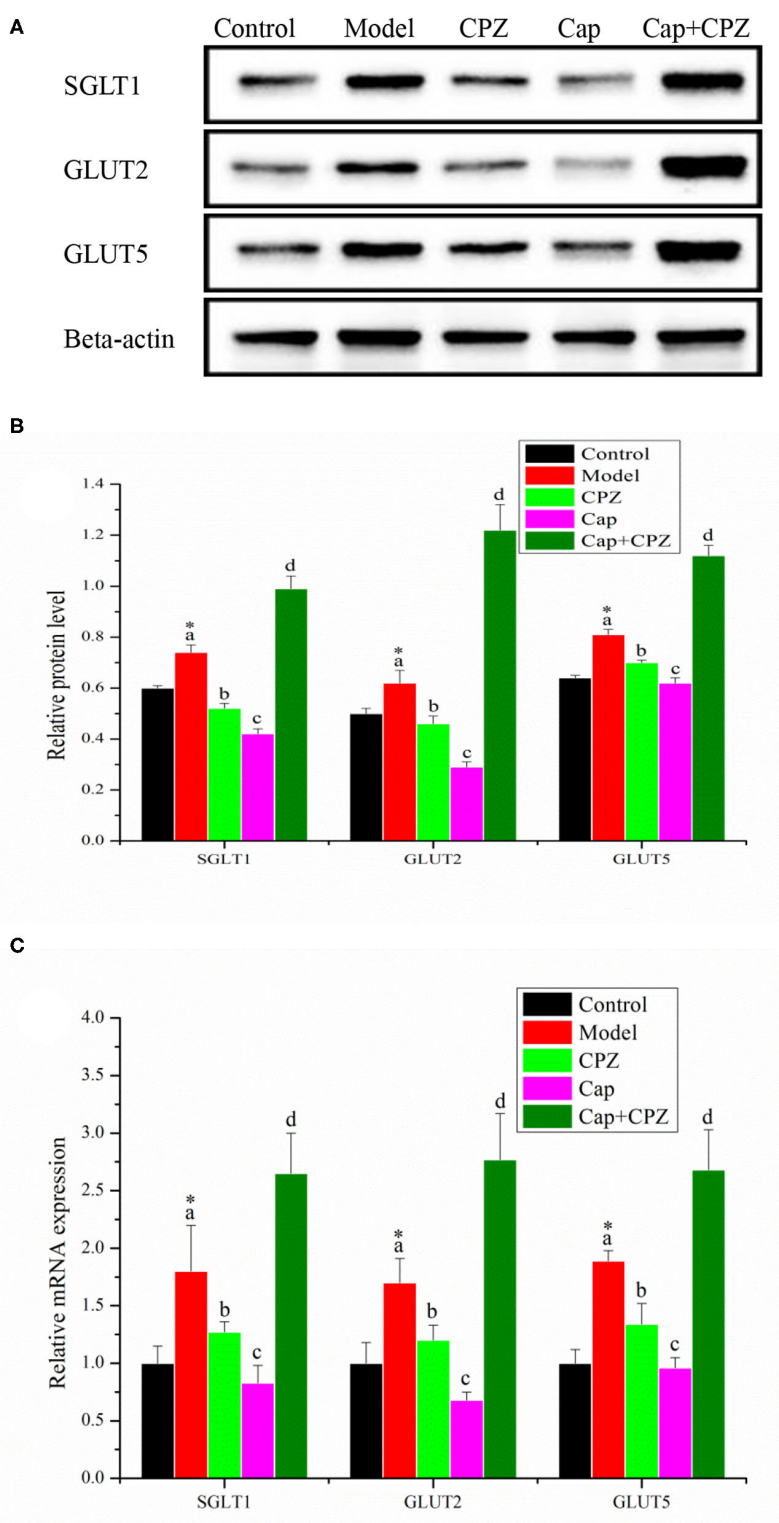
Effects of TRPV1 receptor antagonist and capsaicin on protein **(A,B)** and mRNA **(C)** expression levels of key genes for glycometabolism in ileum of STZ-induced diabetic rats. All data are expressed as means ± SD, *n* = 8. ^*^Means are significant difference compared with the control group (*P* < 0.05). Means with different superscript letters are significantly different among the diabetes groups (*P* < 0.05). SGLT1, sodium/glucose cotransporter 1; GLUT2, glucose transporter 2; GLUT5, Glucose transporter 5. Effective concentrations of capsaicin and capsazepine are 6 and 10 mg/kg·BW, respectively.

As shown in Figures 6A–C, in the model group, the mRNA and protein expression levels of SGLT1, GLUT2, and GLUT5 were significantly up-regulated compared with the control group. After 4 weeks of treatment with capsaicin and capsazepine, the mRNA and protein expression levels of SGLT1, GLUT2, and GLUT5 in the CPZ and Cap groups were significantly down-regulated compared with those in the model group, and the Cap group was the most significant; in the Cap + CPZ group, the mRNA and protein expression levels of SGLT1, GLUT2, and GLUT5 were significantly up-regulated.

## Discussion

In this study, the TRPV1 receptor antagonist (capsazepine) was used to block the activation of the TRPV1 ion channel by capsaicin. The effects of capsazepine, capsaicin, and a combination treatment of capsaicin and capsazepine on a series of biochemical indicators and related genes in STZ-induced diabetic rats were compared. The results showed that the intraperitoneal injection of capsazepine had no significant effect on the body weight, feed intake, TS apparent absorptivity, fasting blood glucose, oral glucose tolerance, insulin level, glycosylated serum protein, or glycogen content in STZ-induced diabetic rats. Capsaicin can significantly reduce TS apparent absorptivity and fasting blood glucose, as well as increase insulin and glycogen content, and repair damaged islet cells in STZ-induced diabetic rats. However, a combined treatment of capsaicin and capsazepine significantly weakened the effect of capsaicin on the above indexes. These results indicate that TRPV1 receptors are involved in the effects of capsaicin on glycometabolism disorder in diabetic rats and are indispensable genes in the mechanism of lowering the glucose level. This observation is consistent with reported studies in which the activation of TRPV1 plays an important role in insulin secretion and islet autoimmune inflammation (11, 23–25). For example, TRPV1+ sensory neurons can control beta cell stress and islet inflammation in autoimmune diabetes (26, 27); the insulin secretion levels of TRPV1 knockout mice were shown to be significantly lower than those of control mice (25); capsaicin can stimulate the insulin secretion of pancreatic islet beta cells by activating TRPV1, and serum insulin was significantly increased by a subcutaneous injection of capsaicin (10 mg/kg) in rats (7).

In order to further clarify the mediating role of TRPV1 receptors in capsaicin-regulated glycometabolism in STZ-induced diabetic rats, the mRNA and protein levels of key genes in the liver, pancreas, and ileum were measured. The results showed that capsaicin up-regulated the expression of GLUT2 and GK, down-regulated the expression of PEPCK and G6Pase, and thus improved gluconeogenesis and glycolysis in the glycometabolism of diabetic rats in order to regulate the level of blood glucose. In the Cap group, the TRPV1 ion channel was activated by capsaicin, the expression of TRPV1 and PDX-1 in the liver and pancreas of diabetic rats was significantly up-regulated, and the hypoglycemic effect was obvious (Figures 4, 5). In the Cap+CPZ group, when the TRPV1 ion channel was blocked by capsazepine, the expression of TRPV1 and PDX-1 in the liver and pancreas of diabetic rats was only marginally increased and capsaicin lost its hypoglycemic effect (Figures 4C, 5C). This indicates that the activation of the TRPV1 ion channel is essential to the hypoglycemic effect of capsaicin. However, when capsiate was administered to diabetic rats, the TRPV1 ion channel was activated while the expression of TRPV1 and PDX-1 in the liver and pancreas of diabetic rats was up-regulated to a certain extent, but the hypoglycemic effect of capsiate was not significant (19). This phenomenon indicates that the activation of the TRPV1 ion channel alone is not enough to lead to a hypoglycemic effect. As shown in Figure 5B, when the TRPV1 ion channel was activated by capsaicin, the protein levels of TRPV1 and PDX-1 were significantly up-regulated by 1.95 and 91 times in the pancreas, respectively, in comparison with the model group. Considering our previous experimental results, when the TRPV1 ion channel was activated by capsiate, the protein levels of TRPV1 and PDX-1 were only up-regulated by 1.3 times and 1 time in the pancreas, respectively (19). Meanwhile, the protein levels of TRPV1 and PDX-1 in the liver also showed a similar phenomenon. Based on these observations, we can propose that capsaicin needs to meet two conditions at the same time to have a significant hypoglycemic effect: (1) activation of the TRPV1 ion channel; (2) significant up-regulation of PDX-1 expression. The hypoglycemic effect of capsaicin can be achieved only when both of the above two conditions are met. However, the specific mechanism of TRPV1-induced PDX-1 up-regulation was not clear in the present study. Relevant studies have shown that continuous infusion of GLP-1 increases PDX-1 expression in rat pancreas (28), and GLP-1 analog liraglutide induces PDX-1 expression in mice (29). TRPV-1 activation increases the release of GLP-1 from L cells in wild type mice, but not in TRPV1^−/−^ mice (25). These results suggest that capsaicin-induced PDX-1 expression is mediated by TRPV-1 activation-GLP-1 release-PDX-1 up-regulation in beta cells. However, to clarify this speculation, more indicators need to be included and more sophisticated methods are needed for further research. For example, measuring plasma GLP-1 levels in STZ diabetic rats and using GLP-1 receptor antagonists would be helpful.

It is well-known that diabetes is an endocrine and metabolic disease characterized by glycometabolism disorders (30, 31). However, when studying the hypoglycemic mechanism of capsaicin, most researchers have focused on the metabolism of lipids in the body instead of the hypoglycemic effect of capsaicin on glycometabolism in the liver, pancreas, and ileum of the body (27–29). As diabetes is highly associated with pancreatic function, it is extremely important to study how capsaicin regulates glycometabolism in the pancreas. As addressed in our experiments and shown in Figure 5, when the TRPV1 ion channel was activated by capsaicin, the expression of TRPV1 receptors and PDX-1 were both significantly up-regulated. PDX-1 further regulated the downstream key genes GLUT2, IRS1, and IRS2. The significant up-regulation of GLUT2 promoted glycogen synthesis and the up-regulation of IRS1 and IRS2 simulated insulin secretion, and consequently, a hypoglycemic effect was achieved. In addition to promoting the secretion of insulin, it is well-documented that PDX-1 can also regulate the proliferation and apoptosis of islet beta cells, as well as promote the transformation of pancreatic ductal cells into islet beta cells (15, 30). Another research study proved that the up-regulation of PDX-1 could not lead to the proliferation of beta cells, but only simulated pancreatic beta cells to secrete more insulin (31). Our previous research results also indicate that appropriate amount of capsaicin (50–100 μmol/L) can stimulate pancreatic beta cells to secrete insulin, but does not have a proliferation effect on islet cells (32), and has a repair effect on damaged pancreatic tissue (Figure 3). It was further confirmed that hypoglycemic effect of capsaicin derived from the stimulation of normal islet cells and repair function of dysfunction cells, rather than the proliferation of islet cells. Therefore, we proposed the TRPV1-mediated hypoglycemic mechanism as the following signaling pathways, TRPV1–(PDX-1)–(GLUT2/GK), and TRPV1–(PDX-1)–(IRS1/2), present in the pancreas, which contributed to the hypoglycemic effect of capsaicin.

In addition, there is an interesting observation in this study that deserves our attention. The administration of combined capsaicin and capsazepine had a negative effect on the intestinal tract of diabetic rats. As shown in Table 1, the apparent absorptivity of TS in the Cap group was significantly reduced, and this is believed to be beneficial for both the hypoglycemic effect and kidney function (reducing the burden of kidney urination). But the apparent absorptivity of TS in the Cap+CPZ group surprisingly increased, indicating that glucose absorption, and metabolism in the intestinal tract of diabetic rats was disordered. This assumption was verified by the determination of relevant genes in the ileum, as shown in Figures 5B,C. When capsazepine blocked the TRPV1 ion channel, in the Cap+CPZ group, the mRNA and protein expression levels of SGLT1, GLUT2, and GLUT5 were significantly increased in the ilium of diabetic rats, indicating that capsaicin combined with capsazepine could lead to a more severe intestinal glycometabolism disorder in STZ-induced diabetic rats and further accelerate the progression of diabetes. Unfortunately, the mechanism causing this phenomenon escaped this study and still needs further research.

## Conclusion

In general, the mediation of TRPV1 receptors in the hypoglycemic effect of capsaicin was studied from the perspective of glycometabolism. The blocking of the TRPV1 ion channel by capsazepine helped verify that the activation of the TRPV1 ion channel was the necessary initial step for capsaicin's hypoglycemic effect. The changes in the expression of key genes TRPV1 and PDX-1 in the pancreas indicated that after the TRPV1 ion channel was activated, the significantly up-regulated expression of TRPV1 receptors and PDX-1 led to a hypoglycemic effect. The signaling pathways in the pancreas were TRPV1-(PDX1)-(GLUT2/GK) and TRPV1-(PDX-1)-(IRS1/2). Overall, the hypoglycemic effect of capsaicin is attributed to three mechanisms: (1) stimulation of pancreatic cells to secrete insulin through signaling pathways TRPV1-(PDX1)-(GLUT2/GK) and TRPV1-(PDX-1)-(IRS1/2); (2) inhibition of the intestinal absorption of glucose and promotion of glycogen synthesis; (3) reduction of the apparent absorption rate and increase of the discharge of sugar in feces.

## Data Availability Statement

The original contributions presented in the study are included in the article/supplementary material, further inquiries can be directed to the corresponding authors.

## Ethics Statement

The animal study was reviewed and approved by Committee on Animal Experimentation [experimental animal license SCXK (Chongqing) 200120008].

## Author Contributions

All authors listed have made a substantial, direct and intellectual contribution to the work, and approved it for publication.

## Funding

This work was supported by the National Natural Science Foundation of China [NSFC 31471581], Special Fund for Outstanding Talented Young and Middle-aged Persons of Lingnan Normal University (Grant No. ZL1817) and Natural Science Foundation of Chongqing Science and Technology Commission (cstc2019 jcyj-msxmX0785).

## Conflict of Interest

The authors declare that the research was conducted in the absence of any commercial or financial relationships that could be construed as a potential conflict of interest.

## Publisher's Note

All claims expressed in this article are solely those of the authors and do not necessarily represent those of their affiliated organizations, or those of the publisher, the editors and the reviewers. Any product that may be evaluated in this article, or claim that may be made by its manufacturer, is not guaranteed or endorsed by the publisher.
